# Emerging RUNX2-Mediated Gene Regulatory Mechanisms Consisting of Multi-Layered Regulatory Networks in Skeletal Development

**DOI:** 10.3390/ijms24032979

**Published:** 2023-02-03

**Authors:** Hironori Hojo

**Affiliations:** Center for Disease Biology and Integrative Medicine, Graduate School of Medicine, The University of Tokyo, Tokyo 113-8655, Japan; hojo@g.ecc.u-tokyo.ac.jp

**Keywords:** Runx2, osteoblast, chondrocyte, skeletal development, gene regulatory network, cis-regulatory element, next-generation sequencer, chromatin accessibility

## Abstract

Skeletal development is tightly coordinated by chondrocytes and osteoblasts, which are derived from skeletal progenitors, and distinct cell-type gene regulatory programs underlie the specification and differentiation of cells. Runt-related transcription factor 2 (Runx2) is essential to chondrocyte hypertrophy and osteoblast differentiation. Genetic studies have revealed the biological functions of *Runx2* and its involvement in skeletal genetic diseases. Meanwhile, molecular biology has provided a framework for our understanding of RUNX2-mediated transactivation at a limited number of cis-regulatory elements. Furthermore, studies using next-generation sequencing (NGS) have provided information on RUNX2-mediated gene regulation at the genome level and novel insights into the multiple layers of gene regulatory mechanisms, including the modes of action of RUNX2, chromatin accessibility, the concept of pioneer factors and phase separation, and three-dimensional chromatin organization. In this review, I summarize the emerging RUNX2-mediated regulatory mechanism from a multi-layer perspective and discuss future perspectives for applications in the treatment of skeletal diseases.

## 1. Introduction

Skeletal development is initiated by three origins of cells at distinct positions in mammalian embryos. These cells migrate, are specific to skeletal cell types, and form skeletal elements throughout the body [1]. The facial bones and cranium are derived from the neural crest. The parietal bone, occipital bone, the base of the skull, and axial skeleton are derived from the paraxial mesoderm. Finally, the appendicular skeleton originates from the lateral plate mesoderm. Two distinct modes of ossification underlie skeletal formation: intramembranous and endochondral ossification. Skull and facial bones develop through intramembranous ossification, in which condensed mesenchymal cells directly differentiate into bone-forming osteoblasts. The remainder of the skeleton develops via endochondral ossification. In this process, a cartilage mold is first formed by chondrocytes following the condensation of mesenchymal cells and is then replaced by mineralized bone tissues.

Two skeletal cell types, including osteoblasts and chondrocytes, are derived from common skeletal progenitors during endochondral ossification. Cells in the center of the condensed mesenchyme differentiate into proliferating chondrocytes and subsequently into pre-hypertrophic and hypertrophic chondrocytes, followed by the formation of mineralized cartilage. Cartilaginous matrix genes, including collagen type II alpha 1 chain (*Col2a1*), collagen type IX alpha 1 chain (*Col9a1*), collagen type XI alpha 1 chain (*Col11a1*), and aggrecan (*Acan*) are strongly expressed in proliferating chondrocytes, whereas collagen type X alpha 1 chain (*Col10a1*) is specifically expressed in hypertrophic chondrocytes. Hypertrophic chondrocytes also highly express secreted phosphoprotein 1 (*SPP1*, also known as *osteopontin*), matrix metallopeptidase 9 (*MMP9*), and matrix metallopeptidase 13 (*MMP13*).

Osteoblast progenitors exist in the perichondrium, a thin layer of cells that originates in the condensed mesenchyme that is located on the periphery of the cartilage. Once the progenitors receive osteogenic inputs, mainly from the factors secreted by hypertrophic chondrocytes, they are specified into osteoblast precursors. They mature into osteoblasts, which deposit the bone matrix, forming a bone collar [1]. In the osteoblast lineage, alkaline phosphatase (*Alpl*) and integrin-binding sialoprotein (*Ibsp*) are expressed in both osteoblast precursors and osteoblasts. A high expression of type I collagen alpha I (*Col1a1*) and *Spp1* has been detected in osteoblasts. Bone gamma-carboxyglutamate protein (*Bglap*, also known as *osteocalcin*) is a bona fide marker of mature osteoblasts. Notably, some secretory calcium-binding phosphoprotein (*SCPP*) genes, including *Ibsp and Spp1*, are commonly expressed in hypertrophic chondrocytes and osteoblasts, representing the signatures of mineralized tissue [2].

In the past three decades, the molecular mechanisms underlying bone development have been extensively studied. Mouse genetics and next-generation sequencing (NGS) analyses have provided broad insights into the regulatory mechanisms underlying the specification and differentiation of skeletal cell types. In this review, I first provide an overview of our understanding of cis-regulatory networks in skeletal cells and then summarize the actions of trans-activators, particularly focusing on Runt-related transcription factor 2 (Runx2): a master regulator of bone development.

## 2. Overview of Gene Regulatory Networks

Gene expression is controlled by interactions between cis-regulatory elements (CREs) and trans-regulatory factors, including transcription factors (TFs) and transcriptional regulators [3]. CREs are composed of noncoding DNA containing binding sites for TFs or other regulatory molecules. Promoters and enhancers are the most well-understood types of CREs. The promoter recruits basal transcription machinery and RNA polymerase to initiate transcription. Although the promoter is required for transcriptional initiation, it cannot properly achieve the transcription of a set of genes in cells with appropriate timing. Additional DNA regulatory elements and enhancers provide the spatiotemporal regulation of gene expression.

Enhancers are defined as DNA sequences that can mediate the spatial and temporal patterns of gene expression regardless of the orientation and location of the sequence. They function at megabase distances from the transcription start sites (TSSs), as exemplified by the long-range interactions that regulate *Shh* expression [4]. TFs and their cofactors bind to enhancers in a sequence-specific manner. The interactions between TFs and CREs are involved in promoter-enhancer associations, resulting in spatiotemporal target gene expression.

Numerous studies have sought to understand cis-trans regulation in the gene regulatory mechanisms of skeletal cells, with a particular focus on identifying CREs in skeletal cells and TFs that act on those CREs, thereby playing pivotal roles in the specification of skeletal cell types. Regarding CREs, the chondrocyte-specific mouse *Col2a1* intron 1 enhancer was shown to be sufficient for chondrocyte-specific enhancer activity [5]; the hypertrophic chondrocyte-specific *Col10a1* enhancer was identified in regions distal to the TSS [6]. In osteoblast markers, an osteoblast-specific *Col1a1* promoter region was identified [7]; osteoblast-specific CREs were also identified in the *Bglap1* promoters identified as osteocalcin-specific element 1 (OSE1) and OSE2 [8].

Regarding transactivators, a limited number of transcriptional regulators were identified as the master regulators of bone development. The SRY-box-containing gene 9 (SOX9) directly binds to *Col2a1* enhancers in chondrocytes. SP7/OSTERIX is associated with the osteoblast-specific *Col1a1* promoter in osteoblasts [9]. RUNX2 binds to the *Col10a1* enhancer in chondrocytes and OSE2 in osteoblasts [10,11]. Consistent with the specificity of cis-trans regulation, *Sox9* is initially required for the establishment of skeletal progenitors and their subsequent differentiation into chondrocytes [12]. *Sp7* is essential for the specification of osteoblast precursors [9]. *Runx2* is required for both osteoblast specification and chondrocyte hypertrophy [10,13,14,15,16].

**Figure 1:**
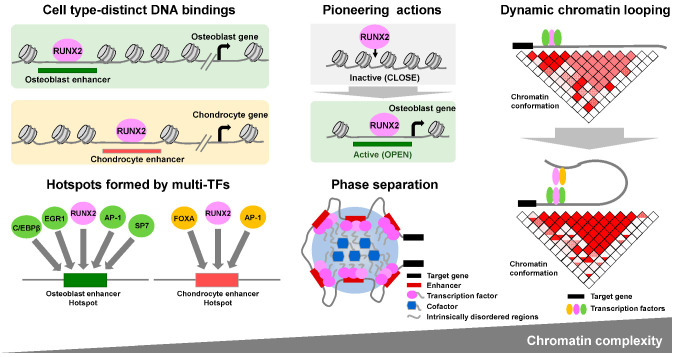
Overview of models of RUNX2-mediated multi-layered gene regulatory mechanisms in skeletal cells.

Overall, as described so far, the frameworks of cis-trans gene regulatory mechanisms in skeletal cell types have been established from the perspective of interactions between a limited number of TFs and specific CREs in marker genes. Furthermore, NGS analysis has extended our understanding of the more complicated gene regulatory mechanisms (Figure 1). Studies have hitherto identified thousands of CREs in given cell types; these CREs are constructed in a three-dimensional manner and dynamically change cell fate specifications and differentiation. TFs form a transcriptional complex and associate with DNA through this complex, some of which actively control the chromatin state itself.

## 3. Cis-Regulatory Networks in Skeletal Cell Types

Cis-regulatory networks in skeletal cell types have been extensively studied by NGS analysis, including chromatin immunoprecipitation sequencing (ChIP-seq): an assay for transposase-accessible chromatin sequencing (ATAC-seq), and RNA sequencing (RNA-seq). Given that some histone modifications represent specific chromatin states [17], combinatorial analysis of several histone modifications provides putative CREs in chondrocytes and osteoblasts. Putative enhancers have been identified in neonatal mouse rib chondrocytes [18], rat chondrocytes [19], human MSC-derived chondrocytes [20], mouse osteoblasts cell lines [21], and human MSC-derived osteoblasts [22]. Some studies have further investigated the dynamics of CREs in osteoblast differentiation by performing a series of ChIP-seq investigations for several histone modifications at different time points in the cell culture [23,24]. Wu et al. proposed that H3K27me3, a histone marker associated with transcriptional repression, was generally lost upon osteoblast differentiation, but this marker was rarely observed in genes undergoing repression during the process, suggesting that transcriptional repression is dominant in progenitor states before induction [23]. Dynamic changes in gene expression were also correlated with H3K36me3, which marks transcriptional elongation, and H3K27ac, which, in turn, marks active enhancers [23].

To identify cell-type distinct CREs in osteoblasts and chondrocytes, we recently published a comparative study of ATAC-seq with sorted cells isolated from cell-type-specific reporter mice at the neonatal stage [25]. This assay provided genome-scale profiles of chromatin-accessible regions where transcriptional regulators could physically associate with DNA [26,27]. We used *Col2a1*-ECFP [28]-positive rib chondrocytes (C2-Cho), *Col10a1*-mCherry [29]-positive hypertrophic chondrocytes, and *Sp7*-EGFP [30]-positive calvaria osteoblasts (*Sp7*-Ob) in newborn mice. Comparative analysis suggested that different repertories of open chromatin regions were used in a cell-type distinct manner. Chromatin accessibility in distal regions (defined as >500 bp away from the transcriptional start site) represented a more cell-type-distinct manner than those in promoter regions. The motif analysis of cell type-specific and distal open chromatin regions revealed a hierarchy of master transcriptional regulators. The Sox9 motif was enriched in open chromatin regions of chondrocytes, whereas the Sp7 motif was enriched in osteoblast-specific regions. The RUNX motif was enriched in hypertrophic chondrocytes and highly enriched in osteoblast-specific open chromatin regions. These data suggest that different CRE repertories are available in distinct cell types, where different combinations of transcriptional regulators may act on distinct repertories.

## 4. Essential Roles of RUNX2 in Skeletal Programs

ChIP-seq for TFs provides genome-scale profiles of TF-DNA binding and the modes of action of TFs. In the past decade, a series of ChIP-seq studies have been performed on skeletal cells, mainly focusing on the master regulators SOX9 in chondrocytes [18,19], SP7 in osteoblasts [31], and RUNX2 in osteoblasts and chondrocytes [21,24,25,32]. In this review, the current understanding of RUNX2 binding signatures is summarized, including a brief review of the biological roles and genome-scale actions of RUNX2 in skeletal cells. This includes recent NGS studies that highlight cell type-distinct and RUNX2-mediated gene regulatory mechanisms.

Runx2 is a transcription factor that belongs to the Runx family, which is composed of Runx1, Runx2, and Runx3. Runx2 is essential for chondrocyte hypertrophy and osteoblast specification [33]. Mutations in the *Runx2* gene have been shown to cause cleidocranial dysplasia [15,34]. Mouse genetic studies have further provided a framework for understanding the roles of Runx2 in osteoblasts and chondrocytes. In the osteoblast program, skeletal progenitors are committed to *Runx2*-positive osteoblast precursors that transition to *Runx2* and *Sp7* double-positive osteoblast precursors before adopting a mature osteoblast phenotype [1]. In *Runx2*-deficient mutant mice, osteoblast differentiation is arrested prior to the activation of *Sp7* [9]. However, *Runx2*-positive osteoblast precursors were present in *Sp7*-deficient mice yet failed to progress to mature osteoblasts [9]. Together, these data suggest that *Runx2* initiates osteogenesis upstream of *Sp7* early in the regulatory hierarchy of osteoblast development.

In the chondrocyte program, *Runx2* is weakly expressed in proliferating columnar chondrocytes but is markedly upregulated as chondrocytes exit the cell cycle, first forming pre-hypertrophic, then hypertrophic chondrocytes. Notably, the ectopic expression of *Runx2* in columnar chondrocytes accelerates chondrocyte hypertrophy [35,36,37], and the removal of *Runx2* activity prevents normal hypertrophic cartilage mineralization [16]. The complete removal of both *Runx2* and *Runx3* results in a complete loss of cartilage and bone mineralization, indicating that *Runx2* and *Runx3* play partially redundant roles in skeletal development and mineralization programs [16]. The restoration of *Runx2* in chondrocytes in *Runx2*-null mutants rescued chondrocyte hypertrophy and mineralization but not the adjacent periosteal bone program. *Runx2* likely acts autonomously and independently of cells in regulating cartilage and bone development [36]. Overall, these genetic studies provide broad insights into the distinct cell type-specific roles of *Runx2* in osteoblasts and chondrocytes.

## 5. Understanding of RUNX2 Association with DNA in Genome-Scale

RUNX2 ChIP-seq studies have provided insights into RUNX2-mediated gene regulation in skeletal cells. In 2014, three independent groups reported RUNX2 ChIP-seq studies on the osteoblast program using in vitro cell cultures [21,22,24]. We recently reported on a RUNX2 ChIP-seq study using primary osteoblasts and chondrocytes isolated from neonatal mouse calvaria and rib cartilage, respectively [25]. These ChIP-seq studies provided the following three major findings: (1) RUNX2 is associated with CREs, (2) the mode of action of RUNX2, and (3) molecular mechanisms of the cell type-distinct roles of RUNX2’s roles.

### 5.1. Runx2 Is Associated with CREs

First, RUNX2 is associated with both proximal and distal genomic regions of osteoblasts [21,22,24]. Distal RUNX2-bound peaks were strongly associated with genes related to skeletal cells and bone development, whereas TSS RUNX2-bound peaks were more strongly associated with general cell activities. The percentage of occupancy in the promoter and distal regions was similar between osteoblasts and chondrocytes [25]. Notably, the binding of RUNX2 to the genome is highly associated with open chromatin signatures in skeletal cells [25]. RUNX2-bound regions in chondrocytes are associated with higher chromatin accessibility in Col10-positive chondrocytes, whereas RUNX2-bound regions in osteoblasts are highly associated with chromatin accessibility in Sp7-positive osteoblasts.

Wu. et al. further showed dynamic changes in RUNX2-bound regions during osteoblast differentiation in vitro [24]. Only a subset of RUNX2 peaks, which were highly associated with osteoblast differentiation-related genes, appeared upon osteoblast induction. Known RUNX2 targets such as *Runx2* itself, *Ibsp*, and *Sp7* are included in this cluster [24]. In contrast, another cluster of RUNX2 peaks, whose intensities are lost upon induction, is related to the biological functions of other cell lineages, including fat cell differentiation, leukocyte migration, and erythrocyte differentiation [24]. These findings suggest that RUNX2 is likely to be broadly associated not only with the distal regulatory regions targeting skeletal genes but also with the TSS regions of genes related to general cell activities in both osteoblasts and chondrocytes.

A recent lineage tracing analysis revealed that hypertrophic chondrocytes could transdifferentiate into osteoblasts [38]. One hypothesis is that genome-scale chromatin reconstruction occurs during transdifferentiation; RUNX2 may contribute to the reorganization of the chromatin landscape, as *Runx2* was recently revealed to be necessary for this process [39]. Further studies focusing on lineage tracing and chromatin dynamics at a single-cell resolution are required to better understand the mechanism underlying genome-scale chromatin reconstruction.

### 5.2. The Mode of Action of RUNX2 (Figure 2)

Second, the primary mode of action for RUNX2 is its binding to the consensus motif of the DNA, regardless of cell type and distance from the TSS [25]. De novo motif analysis showed a high enrichment of Runx consensus motifs in TSS and distal RUNX2 peaks for both osteoblasts and chondrocytes. The high enrichment of the Runx motif is also conserved in human MSC-derived and mouse osteoblasts in vitro [21,22,24]. This mode of action differs from those of the other master regulators, SP7 and SOX9. SP7 acts predominantly on distal enhancers that target osteoblast-specific gene expression [31]. SOX9 associates with both distal enhancers and TSS regions in chondrocytes, although its action on TSS regions is reported to be indirect (via its interaction with the basal transcription complex), as the SOX9 consensus motif is not enriched in the SOX9-associated TSS regions [18].

**Figure 2:**
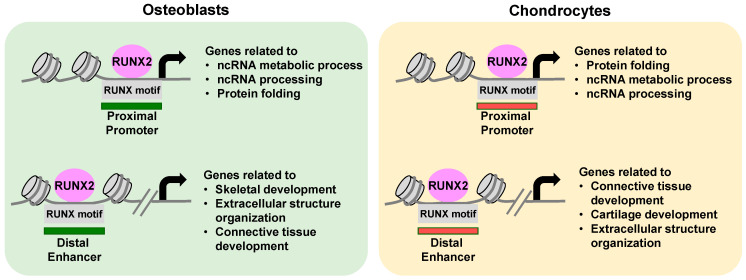
Mode of action of Runx2 in osteoblast and chondrocyte development. Runx2 is associated with TSS regions of genes related to general cell activities; it is also associated with distal cis-regulatory elements targeting distinct cell-type gene sets. Runx2 binding to the consensus motif is the primary mode of action regardless of the distance from TSS. Gene ontology terms enriched by each association modes are shown.

### 5.3. Molecular Mechanisms of the Cell Type-Distinct Roles of RUNX2

**Figure 3:**
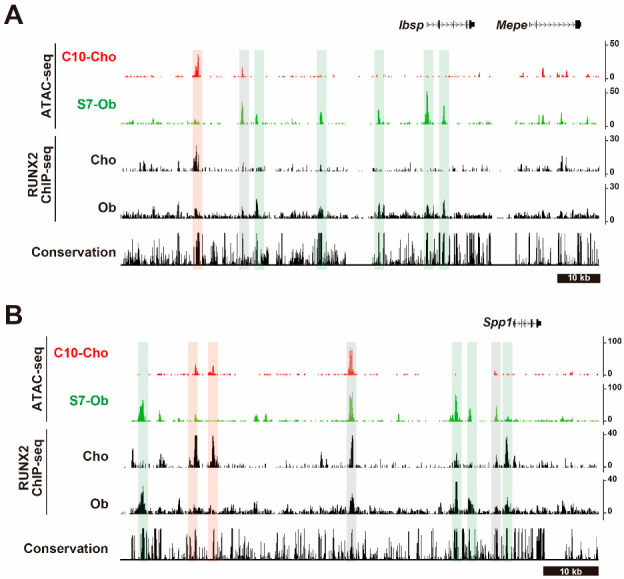
Chromatin accessibilities and RUNX2–DNA binding profiles in the flanking regions of Ibsp and Spp1. CisGenome browser screenshot showing chromatin accessibility and RUNX2–DNA binding profiles in the flanking regions of Ibsp (**A**) and Spp1 (**B**). C10-Cho: ATAC-seq data in sorted cells from Col10 mCherry positive chondrocytes isolated from neonatal rib cartilage in Col10 mCherry reporter mice, S7-Ob: ATAC-seq data in sorted cells from Sp7 EGFP positive osteoblasts isolated from neonatal calvaria bone in Sp7 EGFP mice. Genomic regions highlighted by green color are osteoblast RUNX2 specific regions, those in red are chondrocyte RUNX2 specific regions; those in gray are shared regions in RUNX2 osteoblasts and chondrocytes. These images were visualized by using GEO: GSE178293.

Third, integrative analyses of ChIP-seq for RUNX2 and histone modifications or ATAC-seq for specific skeletal cell types provide putative CREs and target genes regulated by RUNX2 [25]. The analyses not only confirmed RUNX2 binding in the published cis-regulatory elements in *Col10a1* and *Bglap1* described above but also identified a number of new putative targets. Notably, the identification of CREs around SCPP genes suggests a complicated cis-regulatory mechanism in hypertrophic chondrocytes and osteoblasts. Multiple CREs were observed in the genome adjacent to *Ibsp* and *Spp1*, where both shared CREs and cell type-specific CREs were observed (Figure 3) [25]. This suggests that even for commonly expressed genes, the regulatory network differs between osteoblasts and chondrocytes. Despite their similar cellular properties, such as mineralization, distinct gene regulatory landscapes may underlie the biological features of hypertrophic chondrocytes and osteoblasts.

What determines cell type-distinct cis-regulatory networks? Motif analysis integrated with gene expression profiles suggests complex and redundant associations between multiple transcription factors in regulatory regions [25]. In hypertrophic chondrocytes, the FOX motif was exclusively enriched, and *Foxa2*, *Foxa3*, and *Foxc1* were highly expressed, consistent with the crucial rules for chondrogenesis [40,41,42,43]. The SP7-DLX motif [31] was specifically enriched in osteoblasts; *Sp7*, *Dlx3*, *Dlx5*, and *Dlx6* were highly expressed in the cells, which is supported by the fact that these factors are required for osteogenesis [9,44]. However, the common enrichment of AP-1 and ATF motifs with the expression of the corresponding transcription factors in osteoblasts and chondrocytes is consistent with the notion that AP-1 and ATF4 are required for both cell types [45,46,47,48,49]. In addition, other RUNX2 ChIP-seq studies identified C/EBPβ and EGR1 as potential cooperative factors of RUNX2 [21,50]. These studies suggest that not only RUNX2 but also multiple transcriptional regulators are likely to cooperatively regulate with CREs by forming so-called hotspots. Hotspots are defined as genomic regions where key transcriptional regulators co-bind to form core regulatory networks and play a role in specifying cell identity [51,52]. Integrative ChIP-seq analysis for RUNX2 and other transcriptional regulators indicated that several transcriptional regulators associated with genomic regions were correlated with the degree of chromatin accessibility [25]. Among the cis-regulatory elements associated with RUNX2, SP7, and JUN in osteoblasts, the degree of chromatin accessibility was dependent on the number of TFs associated with the regions. This was also the case for cis-regulatory elements bound to RUNX2, JUN, and FOXA2 in chondrocytes. Although only up to three transcription factors were tested in this study, another study showed that the degree of enhancer markers depends on the association of TFs with up to 15 factors in adipogenesis [53].

In addition, putative regulatory networks were drawn based on the integrative analysis, including ChIP-seq for TFs and histone modifications and RNA-seq [41,54,55]. Several MSCs transcriptional factors, including MEF2A and SMAD3, have been identified that could drive osteogenesis and suppress adipogenesis [55]. SOX9-GLI-FOXA phasic regulatory networks have been proposed to be involved in chondrocyte development [42]. However, the role of Runx2 in these regulatory networks remains unclear. Thus, fully understanding this transcriptional complex which contains many transcriptional regulators in skeletal cells, requires further analysis, including omics and especially proteomics studies. Further, gain- or loss-of-function analyses are needed to assess the requirements of combinatorial transcription factors for chromatin accessibility.

In addition to the cooperative actions of RUNX2 and other transcription factors on the genome, several transcriptional regulators have been identified as interacting partners for RUNX2 at the protein–protein level. For example, the core-binding factor β (CBFβ) has been identified as a RUNX cofactor. It does not bind to DNA but increases the transactivation of RUNX2 through a protein–protein interaction, stabilizing the RUNX2 protein [56,57]. In addition to CBFβ, several transcriptional regulators have been identified as cooperative factors of RUNX2 in vitro and in vivo studies, including TWIST [58], STAT1 [59], SCHNURRI3 [60], SATB2 [61], TAZ [62], MED23 [63], NELL-1 [64], and ZFP521 [65]. MicroRNAs and long non-coding RNAs are also involved in Runx2-related gene regulatory networks in skeletal cells [66,67]. Further interaction studies using genome-wide approaches can help clarify their biological relevance from the viewpoint of chromatin accessibility and the gene regulatory landscape. How the post-transcriptional modifications of Runx2 affect the binding properties of genomic DNA and whether any changes are observed in the binding regions or mode of action in the genome remain to be clarified.

## 6. The Actions of RUNX2 in Chromatin Accessibility (Figure 4)

**Figure 4:**
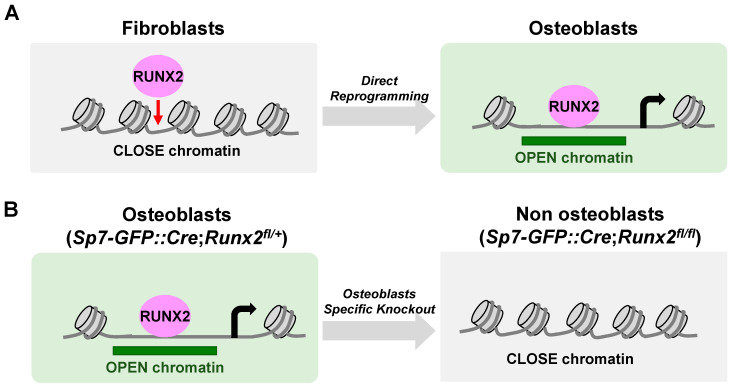
Dynamics of chromatin accessibility by alteration of Runx2 expression. (**A**) Exogenous Runx2 expression enhances chromatin accessibilities via osteoblast direct reprogramming. (**B**) Runx2 ablation using Sp7-GFP::Cre with Runx2-flox decreases chromatin accessibility in the neonatal osteoblasts of mice.

Some transcription factors are not only passively associated with chromatin-accessible regions but are also actively associated with changing chromatin states. For example, a model of “pioneer factors” has been proposed to facilitate the opening of closed chromatin sites [68]. In this model, pioneer factors bind to the inaccessible genomic regions and recruit other TFs, cofactors, and chromatin remodelers to make the underlying DNA more accessible to transcriptional machinery and initiate cell type-specific gene expression programs. Several lines of evidence support the notion that RUNX2 has a pioneer action in osteoblast specification [25] (Figure 4). First, the exogenous expression of *Runx2* in fibroblasts showed DNA binding in closed chromatin regions where chromatin accessibility was later gained. The high enrichment of the consensus RUNX motif in the altered genomic regions upon *Runx2* expression supports the idea that RUNX2 directly associates with genomic sites. Second, a mouse genetic study revealed abundant changes in chromatin accessibility by *Runx2* ablation in Sp7-positive osteoblast precursors. The significantly decreased genomic regions in chromatin accessibility upon *Runx2* ablation were located in regions distal to the TSS, enriched with the RUNX motif, and highly associated with genes related to skeletal system development. These results suggest that RUNX2 was required for chromatin accessibility in osteoblasts, which may underlie the RUNX2-mediated regulatory mechanisms in osteoblast specification.

Given that the pioneering actions of a transcription factor depend on the biological content and presence of a cofactor [69], it remains unclear how RUNX2 acts as a pioneer factor. It also remains to be clarified whether RUNX2 also exerts a pioneer function in chondrocyte hypertrophy and how RUNX2 acts as a pioneer factor. Consistent with RUNX2, RUNX1, and RUNX3 has been suggested to act as pioneer factors in different cell types [70,71]. RUNX1 has been shown to shape the chromatin landscape in metanephric mesenchyme cells and hematopoietic cells [72,73], and RUNX3 was shown to have a pioneering role in cell cycle progression. The Runt domain of RUNX3 physically interacts with the bromodomain of BRD2, which interacts with the MLL1/MLL5 and SWI/SNF protein complexes, promoting chromatin opening [74]. Because the RUNT domain is highly conserved among RUNT members, the domain might be crucial in RUNX2-mediated pioneering actions. Structural analysis of the RUNX2 protein is required to understand how the runt domain is associated with closed chromatin.

## 7. Higher Order Gene Regulation Mechanisms Where RUNX2 Is Involved

Recent studies have indicated that the assembly of the transcription machinery at genomic sites occurs via liquid–liquid phase separation, leading to the formation of transcriptional condensates [75,76]. At these sites, clusters of enhancers are bound by master transcription factors with high densities of coactivators, forming so-called super-enhancers [77]. Indeed, intrinsically disordered regions (IDRs) in the N-terminus of the RUNX2 protein are reportedly involved in phase separation; mutations in the Runx’2 IDRs have been proposed to be involved in cleidocranial dysplasia [78]. Given that phase separation is involved in the transactivation of super-enhancers, RUNX2-mediated organization of chromatin accessibility can be partly accounted for by the formation of hotspots, that is, transcriptional condensates via phase separation. Recent studies further highlight the involvement of RNAs in the formation of phase separation [79]. Thus, the three-dimensional chromatin structure and involvement of combinatorial transcriptional regulators and RNAs in the phase separation should be investigated.

Furthermore, the modeling of three-dimensional chromatin architecture has provided a new framework for understanding gene regulatory networks. Recently developed methods of chromosome conformation capture analyses, such as 4C-seq and Hi-C technology, have revealed that spatiotemporal gene expression is achieved by complicated cis-trans actions from multiple enhancers, and the sum of cell-type- or tissue-distinct activities of each regulatory element leads to the global expression pattern of a gene [80]. A recent Hi-C study integrated with other NGS analyses revealed that a lineage-specific chromatin loop was formed during the differentiation of human MSCs into osteoblasts and adipocytes. One study showed that these lineage-specific loops could activate gene expression and facilitate cell commitment; RUNX2 has been proposed as a key factor in loop-mediated regulatory networks [81].

## 8. Concluding Remarks

NGS studies have provided insights into multiple layers of gene regulatory mechanisms, including TF-DNA binding, histone modification, chromatin accessibility, and three-dimensional chromatin conformation. RUNX2 plays a key role in controlling gene regulatory networks by acting on these various aspects. The remaining points to be clarified are: (1) a better understanding of the involvement of RUNX2 in higher-order chromatin conformation; (2) RUNX2-mediated regulatory networks in the adult skeleton; and (3) cis-regulatory networks underlying human genetics.

Regarding the first point, although RUNX2 is apparently involved in higher-order chromatin organization, the complete picture of gene regulatory mechanisms remains unclear. Knowing which RUNX2 partners are involved in its action and how each layer of gene regulation is coordinated to obtain functional outcomes is vital. Integrative omics analysis, particularly proteomics, will help answer these questions.

Regarding the action of RUNX2 in the adult stage, *Runx2* is required for bone metabolism [82] and also plays multiple roles in pathological conditions such as osteoarthritis [32,83]. As a model of osteoarthritis, we recently reported that RUNX2-DNA binding regions were altered by inflammatory conditions [32]. Understanding the mechanisms underlying these pathological conditions may help identify novel therapeutic targets.

A further understanding of RUNX2-mediated gene regulatory mechanisms may provide several therapeutic aspects. First, as RUNX2-mediated cis-regulatory elements are activated in a cell-type distinct manner [25], enhancers tightly wired with a pathological condition or tissue regeneration should be investigated. Specific enhancers involved in kidney repair were identified in a model of ischemic acute kidney injury [84]; these enhancers can be used as markers of tissue repair. The regeneration-specific enhancer was identified in the zebrafish model [85]. This can be used as a specific gene expression system in the cells that contribute to tissue regeneration [86]. Second, as previously mentioned, the *Runx2* mutant was reported to be involved in the abnormal formation of phase separation [78]. A further understanding of molecular mechanisms in phase separation may help identify tools that can be used to control phase separation. As phase separation is also associated with tumorigenesis [87], the RUNX2-associated phase separation may also be involved in bone-related tumors and form potential targets for diseases.

Finally, from the perspective of human genetics, extensive GWAS studies have identified links between the human genome and skeletal development or diseases [88,89]. More than 90% of disease-associated loci have been identified outside of protein-coding regions, and enhancers comprise approximately 40% of non-coding regions [90]. Recent integrative analyses of GWAS with studies on higher-order gene regulatory networks have identified effector genes of GWAS loci in bone diseases [88,91,92]. Thus, accumulating knowledge on gene regulatory mechanisms will provide a rich resource to connect regulatory variants with human diseases.

## Data Availability

GEO: GSE178293.
